# Cancer: From a Genetic Disorder to a Systemic Disease

**DOI:** 10.1002/advs.202518885

**Published:** 2025-10-07

**Authors:** Ada Hang‐Heng Wong, Yuming Hu

**Affiliations:** ^1^ Deputy Editor for Advanced Science oncology section John Wiley & Sons Inc. Maryland USA; ^2^ Deputy Editor for Advanced Science and EiC for Advanced Genetics John Wiley & Sons Inc. Beijing 100028 China

As we step into the intelligent era, cancer has evolved from being perceived solely as a genetic disorder to being recognized as a systemic disease. In silico oncology has paved the way to elucidate the complex interplay between genes and the environment. While advanced technologies enabled us to delve deeply into the molecular mechanisms of cancer cells and the tumor microenvironment, more research is needed as new pathways are unraveled during cancer initiation, progression, and metastasis. Future research calls us to dive deeper into the mechanisms of cancer etiology, understand the tumor macroenvironment, decipher the cancer exposomics, adopt a holistic approach to cancer treatment, and explore new clinician‐patient dynamics. The interdisciplinary nature of these future directions necessitates more translational research and increased collaboration across multi‐disciplinary teams. However, it also brings new challenges in the domains of public health, environmental and health policies, and legal frontiers. Hence, the renewed definition of cancer paves for groundbreaking research and exciting discoveries.

## The Gene and the Central Dogma

“Gene” had been an abstract form of inheritance when Gregor Mendel crossed his peas in the 19^th^ century and when Wilhelm Johannsen described the *Mendelian Principles of Inheritance* in 1909.^[^ ^1^ ^]^ The materialization of the *Gene* only occurred when the crystal structure of the DNA^1^ double‐helix was solved by Francis H.C. Crick and James D. Watson (Nobel Prize in Physiology or Medicine in 1962^[^ ^2^ ^]^), despite acknowledgement of Rosalind Franklin’s contribution came decades later^[^ ^3^ ^]^ and is still under debate.^[^ ^4^ ^]^

Proving that DNA is heritable matter in living organisms is essential to the *Central Dogma*,^[^ ^5^ ^]^ which elementary school children know today. To be fair, the dogma has been revised several times: first after the discovery of reverse transcriptase by David Baltimore and Howard Temin (Nobel Prize in Physiology and Medicine in 1975^[^ ^2^ ^]^), and then after the discovery of the catalytic activity of RNA^2^ by Sidney Altman and Thomas R. Cech (Nobel Prize in Chemistry in 1989^[^ ^2^ ^]^). The hereditary nature of epigenetics proposed by Conrad Waddington in 1956^[^ ^6^ ^]^ revolutionized our concept of genetics and inheritance. Fast forward to today, the *Central Dogma* covers the fundamentals of genetics, genomics, epigenetics, epigenomics, transcriptomics, and proteomics.

## From Early Observations to Molecular Insights

The term “cancer” means “crab” in Greek, due to its morphological resemblance as a disease before the hallmarks of cancer were defined and re‐defined.^[^ ^7^, ^8^ ^]^ The connection between cancer and genetic disorder emerged after the discovery of the *Philadelphia* chromosome by Peter Nowell and David Hungerford,^[^ ^9^ ^]^ which was eventually visualized by Janet D. Rowley’s Giemsa banding (G‐banding) experiment to be the result of a translocation between chromosomes 9 and 22.^[^ ^10^ ^]^ Subsequently, the development of fluorescence in situ hybridization (FISH) for karyotyping by Joe Gray and Daniel Pinkel ^[^ ^11^ ^]^ and spectral karyotyping^[^ ^12^ ^]^ enabled the construction of the Mitelman Database of Chromosome Aberrations and Gene Fusions in Cancer by Felix Mitelman (https://mitelmandatabase.isb‐cgc.org/).^[^ ^13^ ^]^

While the discovery of genomic translocations has been proven useful for developing targeted therapies, especially for leukemias that undergo huge genomic translocations to form fusion proteins, the discovery of the oncogenic role of virus and hormone by Peyton C. Rous and Charles B. Huggins (Nobel Laureates in Physiology or Medicine in 1966^[^ ^2^ ^]^), respectively, suggested more avenues for cancer etiology. Indeed, tumor virology took flight after Peter K. Vogt’s ground‐breaking experiment of transforming cells with avian retrovirus, and his discovery of the transforming capability of the v‐*src* gene^[^ ^14^ ^]^ led to a gold rush for oncogenes. However, the independent discovery of the proto‐oncogenes of c‐*src* and c‐*myc* genes by Harold Varmus and J. Michael Bishop (Nobel Laureates in Physiology or Medicine in 1989^[^ ^2^ ^]^) established the cellular origins of the viral oncogenes,^[^ ^15^ ^]^ suggesting that normal cells can develop into tumors through mutagenesis of the proto‐oncogenes. Altogether, these studies paved the foundation for oncogene discovery from tumor‐promoting human papillomavirus (HPV), hepatitis B or C viruses (HBV, HCV), and Epstein‐Barr virus (EBV), among others.

On the other hand, the discovery of specific point mutations of the *p53* tumor suppressor gene by Bert Vogelstein,^[^ ^16^ ^]^ and the autosomal recessive *Rb* gene by Alfred Knudson ^[^ ^17^ ^]^ cemented the theory of cancer as a genetic disorder resulting from genetic mutations.

## Evolution of the Mouse Model for Cancer Studies

After establishing that cancer is a genetic disease, a relevant model was established to study oncogenesis. In order to capture the essence of genetic mutation and the complexity of the living organism, the OncoMouse carrying the *MMTV/v‐Ha‐ras* oncogene was created by Philip Leder’s laboratory, as the first patented genetically engineered mouse model.^[^ ^18^ ^]^ After that, mouse models carrying different oncogenes and tumor suppressors, and combinations of such, have been created.^[^ ^19^ ^]^ Furthermore, the introduction of Cre‐recombinase,^[^ ^20^ ^]^ tissue‐ and site‐specific targeting,^[^ ^21^, ^22^, ^23^ ^]^ and viral delivery of CRISPR^3^ vectors^[^ ^24^ ^]^ enhanced the spatial and temporal controllability of tumorigenesis in vivo. Lastly, the Cre‐inducible prime editor mouse model developed by Tyler Jacks’ laboratory^[^ ^25^ ^]^ enabled simultaneous multi‐gene mutagenesis. Mouse zygote electroporation of CRISPR vectors^[^ ^26^ ^]^ also contributed to the acceleration of transgenic mouse engineering from years to months.

Aside from transgenic mouse models, tumor grafts provide an alternative in vivo model to conduct cancer research. The virtue of the animal grafts is the short turnaround time while preserving the in vivo environment. The limited availability of syngeneic allograft models is complemented by the xenograft models. The humanized mouse models^[^ ^27^ ^]^ overcame the issue of immunodeficiency of traditional xenograft models, whereas organoid culture^[^ ^28^ ^]^ and patient‐derived cell lines^[^ ^29^ ^]^ provide additional tools to study cancer in vitro. Attempts to leverage advances in organ transplant studies^[^ ^30^ ^]^ to reconstruct tumor microenvironments in vitro or *ex vivo* led to the advent of microphysiological systems,^[^ ^31^, ^32^ ^]^ which are mostly co‐culture systems by and large, because of the technical complexity in cellular organization and process synchronization to recapitulate tissue morphology and function.

## In Silico Oncology: the Intelligent Era

Even though mouse models provide an excellent tool to study cancer genetics in vivo, it is not a replica of the human organism, whereas in vitro systems lack physiological conditions such as the complex microenvironment and humoral dynamics in vivo. Hence, digital twins technology^[^ ^33^ ^]^ was proposed. This technology is built upon The Cancer Genome Atlas (TCGA),^[^ ^34^ ^]^ together with its subsequent initiatives such as the Human Cell Atlas (HCA),^[^ ^35^ ^]^ Human Epigenome Atlas (HEA),^[^ ^36^, ^37^ ^]^ and Human Protein Atlas (HPA).^[^ ^38^, ^39^ ^]^ Collectively, these initiatives aim to comprehensively map every ‐ome (Greek ‐ōma) of the *Central Dogma* to provide the informatic foundation for big data analysis driven by artificial intelligence (AI) and rational computational methods altogether. Indeed, the abstraction of cells as computation^[^ ^40^ ^]^ has already opened a door for in silico oncology, such as digital pathology,^[^ ^41^ ^]^ prediction of tumor neoantigens^[^ ^42^ ^]^ or T cell receptor specificity,^[^ ^43^ ^]^ theranostics discovery,^[^ ^44^ ^]^ digital drug screening,^[^ ^45^ ^]^ and therapeutic design.^[^ ^46^ ^]^ These technologies make use of our past knowledge gained through decades of cancer genetics research in combination with today's sophisticated techniques. For instance, in comparison to G‐banding, immuno‐FISH on an imaging flow cytometer achieves high‐throughput karyotyping of specific cell types,^[^ ^47^ ^]^ whereas digital karyotyping can be achieved by shallow whole genome sequencing (sWGS)^[^ ^48^ ^]^ at base precision. Similarly, droplet digital PCR (ddPCR) enabled high‐throughput detection of genetic mutations to monitor treatment response in cell‐free DNA,^[^ ^49^ ^]^ although no test has been approved by the Food and Drug Administration (FDA) for clinical use until now. Moreover, data digitization and automated pipelines enable the streamlined analysis of any form of multi‐faceted data. With the construction of sophisticated ultra‐performance computing clusters (UPCCs) and mega data centers, the transmission, compilation, and storage of multi‐omic data becomes feasible, which in turn, facilitate high‐order, high‐content integrated analysis to mimic the complexity reminiscent of in vivo biological systems.

In silico oncology will not only accelerate basic research but also revolutionize clinical practice. First comes patient stratification by existing health records and non‐invasive diagnostic test(s), the combination of which will provide a cost‐effective way to avoid unnecessary screening of low‐risk patients. The development of point‐of‐care systems and telehealth provision will provide convenient access to patients in remote locations. Next comes rational decision‐making for additional screening and/or treatment plans based on the comprehensive analysis of the germline and disease‐specific information gained through multi‐omic analysis. After that, digital health technologies applied to long‐term disease monitoring can be integrated into electronic health records to facilitate intelligent patient management systems to automatically send health alerts and follow‐up reminders. Lastly, telehealth provides unprecedented opportunities to revolutionize the fundamental concept of multi‐center clinical trials to enable global participation and real‐time tracking. Nevertheless, telehealth has its own challenges, including the lack of harmonized data management systems across healthcare systems, the demand for infrastructure enhancement, public concerns of data privacy, and national concerns of regulatory bodies. Furthermore, technological breakthroughs are required to facilitate multi‐modal and high‐content data acquisition from ever‐increasing multiplex assays to achieve truly intelligent systems to realize pragmatic application of in silico oncology. Nevertheless, development of novel data structures and algorithms may transform our current percept of future digital technologies. Consequently, the phase‐out of the animal model for drug development^[^ ^50^ ^]^ may be the final push needed to accelerate research in the field of in silico oncology.

## Beyond the Central Dogma: New Dimension of the Hallmarks of Cancer

Despite the observation of transforming growth factor beta (TGFβ)‐induced epithelial‐to‐mesenchymal transition (EMT),^[^ ^51^ ^]^ cancer remained perceived as a heritable genetic disorder.^[^ ^52^, ^53^ ^]^ This perception began to shift with the hypothesis of cancer stem cells (CSCs) in acute myeloid leukemia (AML) in 1997^[^ ^54^ ^]^ and a breast cancer model in 2003.^[^ ^55^ ^]^ The discovery of induced pluripotent stem cells (iPSC) by Shinya Yamanaka and Sir John B. Gurdon (Nobel Laureates in Physiology or Medicine in 2012^[^ ^2^ ^]^) further supported the idea of cellular plasticity due to de‐differentiation and trans‐differentiation.^[^ ^56^ ^]^ Tissue plasticity, initially observed in breast cancer,^[^ ^57^ ^]^ was later extended to different cancer types.^[^ ^58^ ^]^ Empirical proof of histological transformation^[^ ^59^ ^]^ finally confirms that cancer is more than a genetic disorder; cancer cells undergo a multitude of epigenetic rewiring, which is disturbed by both their germline and environmental cues. Alternatively, observation of sex differences in cancer incidence and outcomes has been conferred to the sex hormones and sex chromosomes with particular focus in the immune‐oncological domain.^[^ ^60^, ^61^ ^]^ Additionally, cancer metabolism studies have also expanded from glucose and lactate of the Warburg effect^[^ ^62^ ^]^ to other metabolites, such as the amino acids, lipids and their derivatives.^[^ ^63^, ^64^ ^]^ Because of the renewed concept, relationships between physical activity^[^ ^65^, ^66^ ^]^ and nutrition^[^ ^67^ ^]^ with cancer prevention, therapeutic outcome or care are also being actively investigated. Furthermore, studies on how the microbiome may drive carcinogenesis are also gaining momentum.^[^ ^68^, ^69^ ^]^ Collectively, these findings highlighted the new dimension of the hallmarks of cancer – that is, cancer is no longer restricted to genetic mutations and the cancer cells, but also encompasses its microenvironment and microbiota.^[^ ^70^ ^]^ Hence, it remains skeptical if the *Central Dogma* may be revised in light of our growing knowledge of the holistic self and the environment.

**Figure 1:**
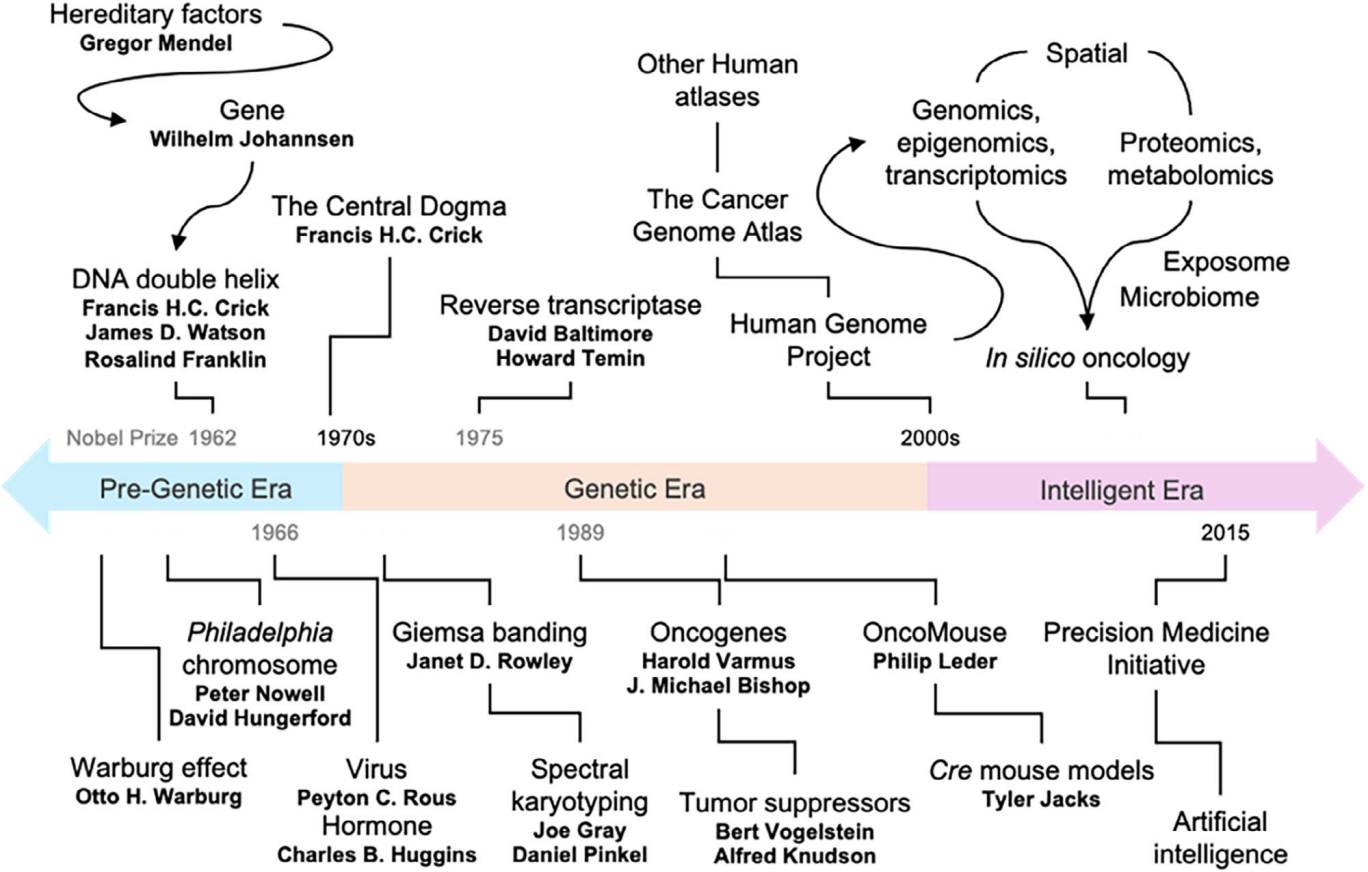
Milestones of cancer research.

Cancer has evolved past the physical and conceptual boundary of DNA and the *Gene* to become a systemic disease (**Figure** **1**). The *Gene* and *Environment* will be the cornerstone of multi‐omics for health. Hence, we call for more research to refine our understanding of genome instability and genetic inheritance with advanced technology, for example, how chromothripsis,^[^ ^71^ ^]^ extrachromosomal DNA (ecDNA),^[^ ^72^ ^]^ and mitochondria transfer^[^ ^73^ ^]^ contribute to carcinogenesis. There is also an unmet need to track single cells longitudinally to elucidate clonal evolution,^[^ ^74^, ^75^ ^]^ trace cell fate,^[^ ^76^ ^]^ and decipher cellular plasticity.^[^ ^77^ ^]^ Furthermore, we need to gain in‐depth insights of the tumor microenvironment at high‐resolution spatial and temporal scales to facilitate the design of precise therapeutic interventions, such as deciphering the spatio‐temporal recruitment of immune cells during cancer eradication or evasion by comparing between responders and non‐responders. This also includes gaining insight into the complexity of the autocrine, paracrine, and endocrine components of the tumor ecosystem on tumorigenesis, progression, metastasis, and drug resistance. Next, understanding the tumor macroenvironment, such as the relationships among nutrition, physiology, and carcinogenesis, is urgently needed to improve treatment outcomes and patient management. Simultaneously, the internal and external exposome should be taken into account to adopt a holistic approach to cancer treatment, leveraging benefits of the abscopal effect,^[^ ^78^ ^]^ enhancing palliative care, and focusing on the patients’ quality of life (QoL). Initiatives such as the Human Exposome Project^[^ ^79^ ^]^ aim to decipher the impact of environmental pollutants and contaminants on carcinogenesis and human health, which will inform regulatory updates for pollution detection and prevention. Comorbidity studies are inevitable to optimize outcomes. Lastly, new clinician‐patient dynamics in the era of precision medicine will require research into effective scientific communication to empower patients to make informed decisions and raise public awareness.

All these efforts can only be achieved with synergistic collaboration among all stakeholders. Leveraging our past achievements, *Advanced Science* and our *Advanced* portfolio are determined to lead this global revolution with our fellow researchers, advancing science for the greater good of humanity.

## Footnotes

^1^
DNA: deoxyribonucleic acid.

^2^
RNA: ribonucleic acid.

^3^
CRISPR: clustered regularly interspaced short palindromic repeats.
